# Autoimmune uveitis attenuated in diabetic mice through imbalance of Th1/Th17 differentiation via suppression of AP-1 signaling pathway in Th cells

**DOI:** 10.3389/fimmu.2024.1347018

**Published:** 2024-06-03

**Authors:** Masaru Takeuchi, Yoshiaki Nishio, Hideaki Someya, Tomohito Sato, Akihiko Yoshimura, Masataka Ito, Kozo Harimoto

**Affiliations:** ^1^ Department of Ophthalmology, National Defense Medical College, Tokorozawa, Saitama, Japan; ^2^ Department of Microbiology and Immunology, Keio University School of Medicine, Tokyo, Japan; ^3^ Department of Developmental Anatomy and Regenerative Biology, National Defense Medical College, Tokorozawa, Saitama, Japan

**Keywords:** experimental autoimmune uveoretinitis, diabetes mellitus, T-cell mediated 34 immunity, AP-1 signaling pathway, Th17 cells, regulatory T cells

## Abstract

**Purpose:**

Inflammation is involved in the pathogenesis of diabetes, however the impact of diabetes on organ-specific autoimmune diseases remains unexplored. Experimental autoimmune uveoretinitis (EAU) is a widely accepted animal model of human endogenous uveitis. In this study, we investigated the effects of diabetic conditions on the development of EAU using a mouse diabetes model.

**Methods:**

EAU was induced in wild-type C57BL/6 (WT) mice and Ins2^Akita^ (Akita) mice with spontaneous diabetes by immunization with IRBP peptide. Clinical and histopathological examinations, and analysis of T cell activation state were conducted. In addition, alternations in the composition of immune cell types and gene expression profiles of relevant immune functions were identified using single-cell RNA sequencing.

**Results:**

The development of EAU was significantly attenuated in immunized Akita (Akita-EAU) mice compared with immunized WT (WT-EAU) mice, although T cells were fully activated in Akita-EAU mice, and the differentiation into Th17 cells and regulatory T (Treg) cells was promoted. However, Th1 cell differentiation was inhibited in Akita-EAU mice, and single-cell analysis indicated that gene expression associated AP-1 signaling pathway (JUN, FOS, and FOSB) was downregulated not only in Th1 cells but also in Th17, and Treg cells in Akita-EAU mice at the onset of EAU.

**Conclusions:**

In diabetic mice, EAU was significantly attenuated. This was related to selective inhibition of Th1 cell differentiation and downregulated AP-1 signaling pathway in both Th1 and Th17 cells.

## Introduction

Diabetes mellitus (DM) is a critical health issue worldwide. It is a consequence of impaired glucose metabolism causing insulin deficiency or resistance, which leads to hyperglycemia and subsequent development of vascular and neuropathic complications. The number of DM patients increases in both developing and developed countries. According to the International Diabetes Federation, the global diabetes prevalence was approximately 9.3% (463 million people) in 2019, and is estimated to increase to 10.2% (578 million) by 2030 and 10.9% (700 million) by 2045 (1). Hyperglycemia arised from insulin deficiency as in type 1 diabetes (T1D) or insulin resistance as in type 2 diabetes (T2D) leads to various clinical complications.

T-helper (Th) cells maintain immune homeostasis in vertebrates, and are polarized into Th1, Th2, Th9, Th17, Th22, T-regulatory (Treg), or follicular helper T (Tfh) cells according to types of cytokines produced under the antigen stimulations in different environments (2, 3). Th17 cells are characterized by expression of the transcription factor retinoic acid receptor-related orphan receptor gamma-T (RORgt), and they produce mainly interleukin (IL)-17 (A-F) (4) as well as IL-21 and IL-22 (5), bridging innate immunity to acquired immunity. Accumulated evidence indicates that dysfunction of Th17 cells contributes to the development of various disorders, and DM is also not the exception (6, 7). Chen et al. reported that the frequency of Th17 cells and IL-17A levels in peripheral blood mononuclear cells (PBMCs) were significantly lower in patients with diabetic retinopathy (DR) than in those without DR, and tended to decrease with increasing DR severity (7). In addition, IL-17 is expressed on pancreatic β-cells from T1D and T2D donors compared with non-diabetic donors or insulin-deficient islets from T1D donors (8). Although the involvement of Th17 cells in the development of T2D remain to be elucidated, Ohshima et al. reported that IL-17 potentially plays a critical role in the pathogenesis of angiotensin II type 1 receptor–induced insulin resistance (9).

The chronic hyperglycemic state increases the risk of several complications caused by macrovascular and microvascular damage and impaired immune function, which especially involve in the brain, kidney, and eyes. DR is one of the most serious complications associated with diabetes. It can lead to vision loss due to progressive damage to the retinal blood vessels and nerves. Additionally, serum IL-17 levels are elevated in patients with DR compared to controls (7, 10, 11), and increased IL-17A level is detected in the ocular fluid of eyes with proliferative diabetic retinopathy (PDR) (12–14). Ins2^Akita^ (Akita) mice have C57BL/6 background with a spontaneous mutation in the insulin 2 gene, resulting in severe insulin-dependent diabetes from 3 to 4 weeks of age (15, 16). In our previous study using Akita mice backcrossed with interferon-γ knock out (GKO) mice where Th cell differentiation toward Th17 cells are promoted (17, 18), VEGF production in the eye and leukostasis in retinal vessels increased significantly in Akita-GKO mice compared with Akita mice (19).

Experimental autoimmune uveoretinitis (EAU), is an organ-specific animal model of human noninfectious uveitis, is able to be developed in various rodent strains by immunization with retinal autoantigens, such as interphotoreceptor retinoid-binding protein (IRBP), or T-cell epitope peptides in complete Freund’s adjuvant (CFA) (20). In the development of EAU, Th1 and Th17 cells play pivotal roles (17, 21), while Treg cells contribute to amelioration of the disease (22–24). One may postulate that if the chronic diabetic state in Akita mice promotes differentiation into Th17 cells, EAU would be exacerbated in Akita mice compared with the wild-type (WT) mice. In this study, we investigated the effects of DM on the development of EAU and the underlying immune mechanisms in Akita mice.

## Materials and methods

### Animals

Eight- to nine-week-old wild-type C57BL/6N (WT) mice and Akita mice with the same background were purchased from Japan SLC Inc. (Shizuoka, Japan). Since male Akita mice have higher blood sugar levels than females and diabetic symptoms are more pronounced (25), we used male mice in this study. All mice were housed in the Center for Laboratory Animal Science of the National Defense Medical College under specific pathogen-free conditions with a regular light-dark cycle (14 h of light and 10 h of darkness per day) and access to food and water *ad libitum*. The study protocols were reviewed and approved by the Animal Ethics Committee of the National Defense Medical College, and the procedures were carried out according to the Association for Research in Vision and Ophthalmology Statement for the Use of Animals in Ophthalmic and Vision Research.

### EAU induction

WT and Akita mice were divided into 2 groups each, intact WT (WT), intact Akita (Akita), hIRBP-p-immunized WT (WT-EAU), and hIRBP-p-immunized Akita (Akita-EAU) mice. EAU was induced in immunized WT and hIRBP-p-immunized Akita mice as described previously with some modifications (26). Briefly, mice were subcutaneously immunized around the neck region with 200 µg of human IRBP 1-20 (hIRBP-p) emulsified in 0.2 mL of complete Freund’s adjuvant (Difco, Detroit, MI, USA) with 1 mg of *Mycobacterium tuberculosis* strain H37Ra (Difco). In addition, 0.5 μg of pertussis toxin (Sigma Aldrich, St. Louis, MO, USA) in 0.2 mL of PBS was injected intraperitoneally.

### Clinical scoring

Clinical scoring of EAU was assessed on days 0, 7, 14, and 21 post-immunization, using a Micron IV fundus camera (Phoenix Research Labs, Pleasanton, CA). Clinical severity was graded on a scale of 0 to 4 according to the previous report (26).

### Histological scoring

Histological study was conducted in WT-EAU and Akita-EAU mice on day 21 post-immunization. The mice were deeply anesthetized with pentobarbital (Kyoritsu, Tokyo, Japan) and isoflurane (Wako, Osaka, Japan), and perfused with 4% paraformaldehyde phosphate buffer solution (Wako) for fixation. The eyes were collected and fixed in the same fixative overnight at 4°C and embedded in paraffin. Sections of 5-µm thickness were prepared and stained with hematoxylin and eosin. Severity of ocular inflammation was score on a scale of 0 to 4 according to the previous report (26).

### Flow cytometry

Cells were collected from the spleen and the draining lymph nodes (DLNs) of intact WT and Akita mice and hIRBP-p-immunized WT and Akita mice on day 21 and were homogenized. Cell suspensions were incubated with anti-mouse CD16/CD32 (eBioscience, San Diego, CA) to block Fc before staining. For detection of activated T cells in spleen, the cells were stained with CD3-APC/Cyanine7 (clone:17A2, BioLegend, San Diego, CA), CD4-APC (clone: RM4-5), CD44-FITC (clone: IM7), and CD62L-PE (clone: W18021D) antibodies. For preparation of intracellular staining of cytokines, the cells were resuspended in complete RPMI1640 medium (Wako) supplemented with 10% fetal bovine serum (Gibco, Waltham, MA), 1 mM sodium pyruvate (Gibco), 1% nonessential amino acids (Gibco), 100 unit/mL penicillin and 100 µg/mL streptomycin (Gibco), and 50 µM 2-mercaptoethanol (Wako), and then stimulated with 50 ng/mL phorbol 12-myristate 13-acetate (Sigma-Aldrich, St. Louis, MO) and 0.5 µg/mL ionomycin (Sigma-Aldrich) in the presence of 10 µg/mL brefeldin A (Sigma-Aldrich) for 4 h at 37°C. After incubation, the cells were collected, and T cell markers were stained with CD3-Pacific Blue (clone 17A2) and CD4-PerCP/Cyanine5.5 (clone GK1.5) antibodies. For intracellular staining, the cells were fixed, treated by permeabilization/fixation buffer [Becton Dickinson (BD) Bioscience, Franklin Lakes, NJ] according to the manufacturer’s instructions, and incubated with IFN-γ-APC (clone XMG1.2) or IL-17A-APC (clone TC11-18H10.1) antibody. For staining of Foxp3, after incubation with CD3-APC/Cyanine7 and CD4-APC antibodies, cells were stained with Foxp3-Brilliant Violet 421 (clone MF-14) antibody using Foxp3/Transcription Factor Staining Buffer Set (eBioscience). After staining, the cells were analyzed by FACSCanto II Flow Cytometer (BD), and the data were analyzed by CellQuest software (BD). The gating strategies for Th cells and CD44^+^/CD62L^+^ T cells are shown in **Supplementary Figure S1**.

### Quantitation of cytokines

Cell suspensions from spleens were cultured with 0 or 10 µg/mL hIRBP-p for 72 h in complete RPMI1640 (cRPMI 1640) medium (27). After incubation, the supernatants were collected and assayed for cytokines (IL-6, IL-10, IL-17, IFN-γ and TNFα) using the Bio-Plex Multiplex Immunoassay System (Takara Bio, Shiga, Japan).

### Single-cell RNA sequencing

#### Sample preparation

On day 21 post-immunization, the mice were anesthetized, and their spleens were obtained. To enhance the purity of T cells, the spleen cell suspension was sorted using the magnetic-activated cell sorting (MACS) cell separator system with the Pan T cell isolation kit (Miltenyi Biotec, Bergisch Gladbach, Germany). Samples were frozen and stored in liquid nitrogen until before use.

#### scRNA-seq data processing

The single-cell suspensions were transformed into barcoded scRNA-seq libraries using the Chromium Single Cell 3′ Library (10X Genomics, Genomics chromium platform Illumina NovaSeq 6000), Chip Kit (10X Genomics), Gel Bead and Multiplex Kit. The quality of the libraries was checked by FastQC software. The CellRanger software (version 6.1.2; 10X Genomics) was employed for the preliminary processing of the Sequencing data. The count pipeline was applied to demultiplex and barcode the sequences. According to the single-cell expression matrix calculated by CellRanger, cells with fewer than 200 detected genes and a mitochondrial gene ratio greater than 20% were removed. Finally, a total of 10,594 cells (WT, 1,940 cells; Akita, 2,490 cells; WT-EAU, 2,623 cells; Akita-EAU, 3,541 cells shown in **Supplementary Figure S2**) were analyzed for the subsequent studies, including normalization, dimension reduction, clustering and differential gene expression genes (DEGs) analysis by using Seurat package (version 3.2.2). The R package harmony was employed to remove batch effect.

#### DEGs analysis

DEGs analysis was performed by using the venice test in the ‘‘Differential expression’’ function of the BBrowserX (BioTuring, San Diego, CA) (28). DEGs were identified to adjust *P* values of less than 0.05 and Log2 fold change > 0.25.

#### Gene ontology analysis

BBrowserX was used to perform GO biological process and pathway analysis to visualize the functional patterns of DEGs, and performed statistical analysis using AUCell (29). Finally, we showed 10 GO terms or pathways related to EAU that enriched by Akita-EAU/WT-EAU DEGs comparison group.

### Real-time polymerase chain reaction

Spleen cells were collected from WT and Akita mice on day 21 post-immunization and then purified to obtain a T cell-rich fraction, as described above. Total RNA was extracted from the cells using RNeasy mini kit (Qiagen, Venlo, Netherlands) according to the manufacturer’s instructions. Total RNA was reverse transcribed into cDNA using the PrimeScript RT reagent kit (TaKaRa, Siga, Japan), according to the manufacturer’s instructions. The all probes, Jun (Mm07296811_s1), Fos (Mm00487425_m1), and Rn18s (Mm03928990_g1), were purchased from Applied Biosystems (Foster City, CA). Amplification was performed in a 7900HT Fast Real-Time PCR System (Life Technologies). The following PCR conditions were used: 50°C for 2 min, 95°C for 15 min, 50 cycles at 95°C for 30 sec and 60°C for 1 min, followed by 25°C for 2 min. The expression levels of target genes were normalized to the 18s expression levels.

### Enzyme-linked immunosorbent assay

T cell-rich fractions were obtained from WT and Akita mice on day 21 post-immunization as described previously. Cell lysate was prepared using Radio-Immunoprecipitation Assay (RIPA) Buffer (Wako) supplemented with protease inhibitor cocktail (Sigma-Aldrich). JUN and FOS protein levels in the cell lysates were measured using Mouse JUN ELISA kit and Mouse FOS ELISA kit (Biorbyt, Cambridge, UK).

### Statistical analyses

JMP pro 15 (SAS Institute, Cary, NC) was used for statistical analyses. Fisher’s exact test was used for statistical analysis of the incidence of EAU. Wilcoxon test was used for statistical analyses of the clinical score, histological score, T cell activation, IFN-γ- or IL-17A-producing CD4^+^ T cells, Treg cells, and cytokines in culture supernatant. *P* values less than 0.05 were considered significant.

## Results

### EAU development in Akita mice by immunization with hIRBP-p

**Figure 1:**
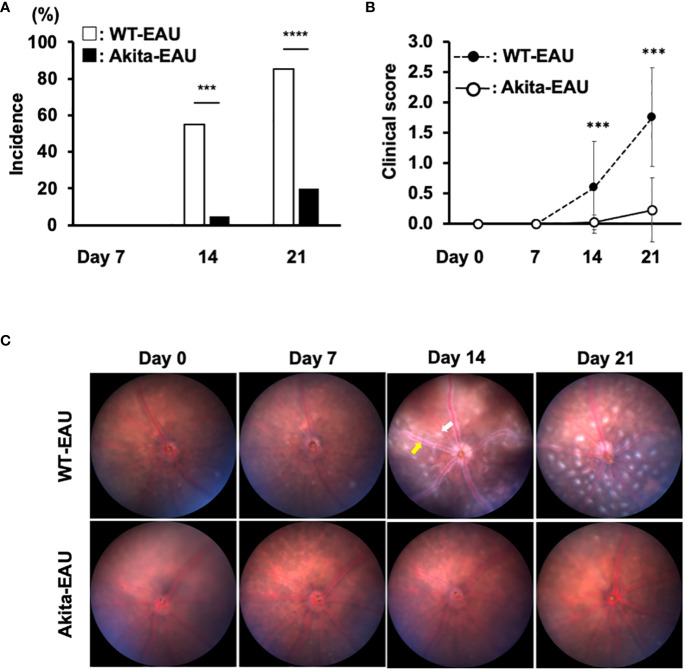
Clinical investigation of EAU in WT and Akita mice immunized with hIRBP-p. **(A)** Incidence of EAU in WT and Akita mice on days 14 and 21 post-immunization is shown. ***p<0.001, ****p<0.0001 determined using Fisher’s exact test. **(B)** Mean clinical scores on days 0, 7, 14 and 21 post-immunization in immunized WT and Akita mice are expressed as mean ± SD for n = 20 eyes in each group. ***p < 0.001 determined using Wilcoxon test. **(C)** Representative color fundus images in WT and Akita mice on days 0, 7, 14 and 21 post-immunization. One example of exudate is indicated by the white arrow, and vasculitis is indicated by the yellow arrow. Data are representative of three independent experiments with similar results.

**Figure 1** presents the clinical incidence and severity of EAU induced by immunization with hIRBP-p in WT mice and Akita mice. EAU developed from day 14 after immunization in both groups, but the incidence was significantly lower in Akita-EAU mice than in WT-EAU mice (**Figure 1A**). WT-EAU mice developed EAU in 11 of 20 eyes (55%) on day 14 and 17 of 20 eyes on day 21 (85%), while Akita-EAU mice developed EAU in 1 of 20 eyes (5%) on day 14 and 4 of 20 eyes (20%) on day 21. Mean clinical score of EAU was significantly higher (more severe) in WT-EAU mice than in Akita-EAU mice both on day 14 (0.6 ± 0.72 vs. 0.03 ± 0.11) and on day 21 (1.75 ± 0.91 vs. 0.23 ± 0.53) (**Figure 1B**). Representative color fundus images show multiple retinal exudates and extensive retinal vasculitis on day 14 and increased retinal exudates on day 21 in a WT-EAU mouse (**Figure 1C**). On the other hand, multiple retinal exudates and extensive retinal vasculitis were much milder in an Akita-EAU mouse compared with a WT-EAU mouse on both days 14 and 21.

**Figure 2:**
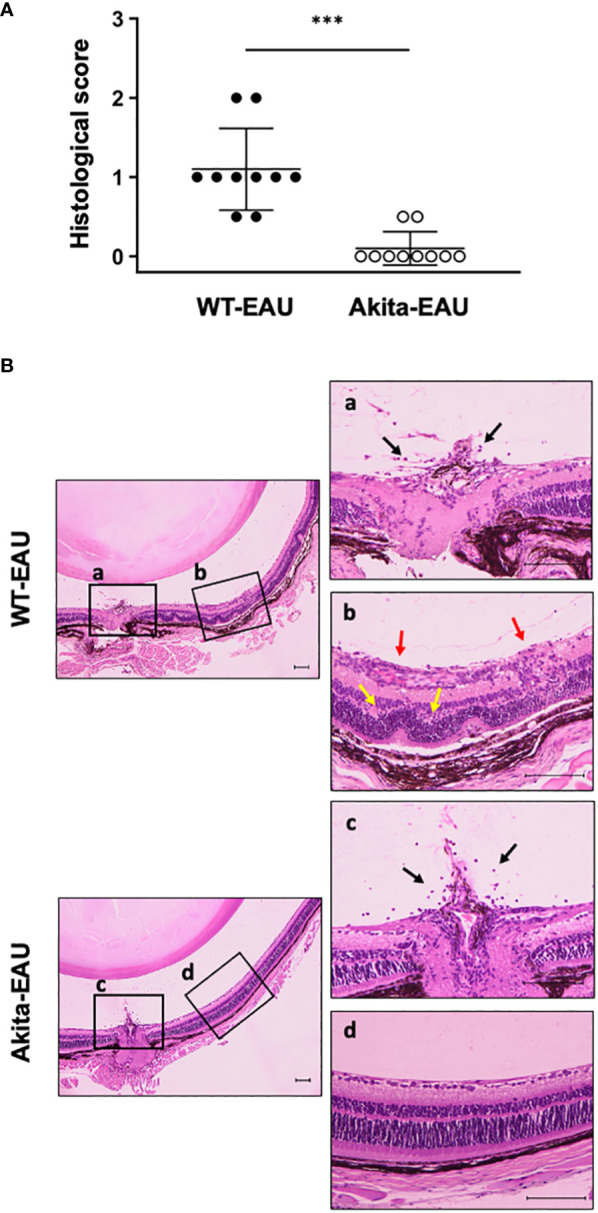
Histopathological evaluation of EAU in WT and Akita mice. **(A)** Mean histological scores on day 21 post-immunization in WT and Akita mice are expressed as mean ± SD for n = 10 sections in each group. ***p < 0.001 determined using Wilcoxon test. **(B)** Representative photomicrographs of histological sections from immunized WT and Akita mice. The boxed areas (a-d) are shown on the right at higher magnification. Histopathological findings such as infiltrating cells (black arrows), vasculitis (red arrows) and destruction of the retinal layer structure (yellow arrows) are observed. *Scale bars*: 100 µm. Data are representative of three independent experiments with similar results.

Histological evaluation of EAU in WT-EAU and Akita-EAU mice on day 21 after immunization with hIRBP-p are displayed in **Figure 2**. Development of EAU was observed in all 10 eyes (100%) of WT-EAU mice, while the incidence was reduced to 2 of 10 eyes (20%) in Akita-EAU mice. Mean histopathological score was significantly higher in WT-EAU mice (1.11 ± 0.52) than in Akita-EAU mice (0.10 ± 0.21) (**Figure 2A**). Representative histopathological micrographs of the eye of a WT-EAU mouse showed cells infiltrating the entire layers of the retina, severe retinal vasculitis, destruction of the retinal layer structure, and partial loss of the outer retinal layers, while the micrographs of the eye of an Akita-EAU mouse showed only mild cell infiltration in the vitreous and the retina (**Figure 2B**).

### T cell states in Akita mice immunized with hIRBP-p

**Figure 3:**
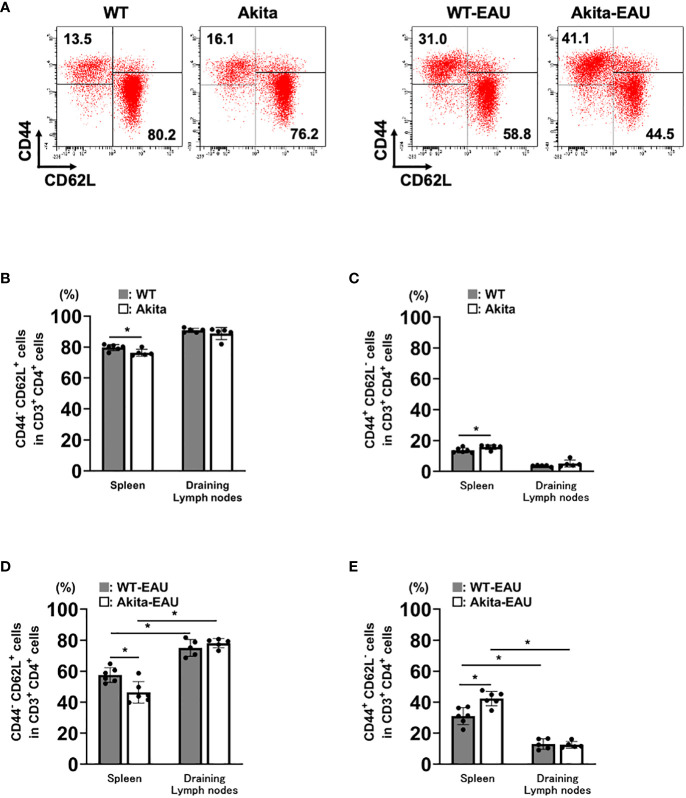
T cell activation in the spleen and the draining lymph nodes (DLNs) of Akita mice immunized with hIRBP-p. Cells were obtained from the spleen and DLNs of intact WT and Akita mice and hIRBP-p-immunized WT and Akita mice on day 21, and were analyzed for expression of CD3, CD4, CD44, and CD62L by flow cytometer. **(A)** The representative flow cytometry plots. **(B)** CD44^-^CD62L^+^ cells and **(C)** CD44^+^CD62L^-^ cells in CD3^+^CD4^+^ T cells obtained from the spleen or the DLNs of intact WT and Akita mice, and **(D)** CD44^-^CD62L^+^ cells and **(E)** CD44^+^CD62L^-^ cells in CD3^+^CD4^+^ T cells obtained from the spleen or the DLNs of hIRBP-p-immunized WT and Akita mice are expressed as mean ± SD for n = 5-6 mice in each group. *P < 0.05 determined using Wilcoxon test. Data are representative of three independent experiments with similar results.

The activation of T cells via the recognition of antigens is essential for the development of EAU. We investigated whether immunization with hIRBP-p resulted in the sufficient sensitization of IRBP-specific T cells in Akita mice. **Figure 3** shows the expression of CD44 and CD62L among CD3^+^CD4^+^ T cells obtained from the spleen and the DLNs in WT and Akita mice with or without hIRBP-p immunization. The representative flow cytometry plots are exhibited in **Figure 3A**. The proportions of naïve T cells expressing CD44^-^CD62L^+^ was higher than activated T cells expressing CD44^+^CD62L^-^ in the spleen and the DLNs of intact WT and Akita mice (**Figures 3B, C**). In the spleen, naïve T cells were significantly fewer and activated T cells were more in Akita mice than in WT mice. Immunization with hIRBP-p resulted in decrease in naïve T cells with CD44^-^CD62L^+^ and increase in activated T cells with CD44^+^CD62L^-^, which were greater in the spleen than in the DLNs (**Figures 3D, E**). The proportion of naïve T cells was significantly lower and that of activated T cells was higher in the spleen than in the DLNs in both WT-EAU and Akita-mice. In addition, naïve T cells were significantly fewer and activated T cells were more in Akita-EAU mice than in WT-EAU mice in the spleen.

### Frequencies of Th1, Th17, and regulatory T cells in hIRBP-p-immunized Akita mice

**Figure 4:**
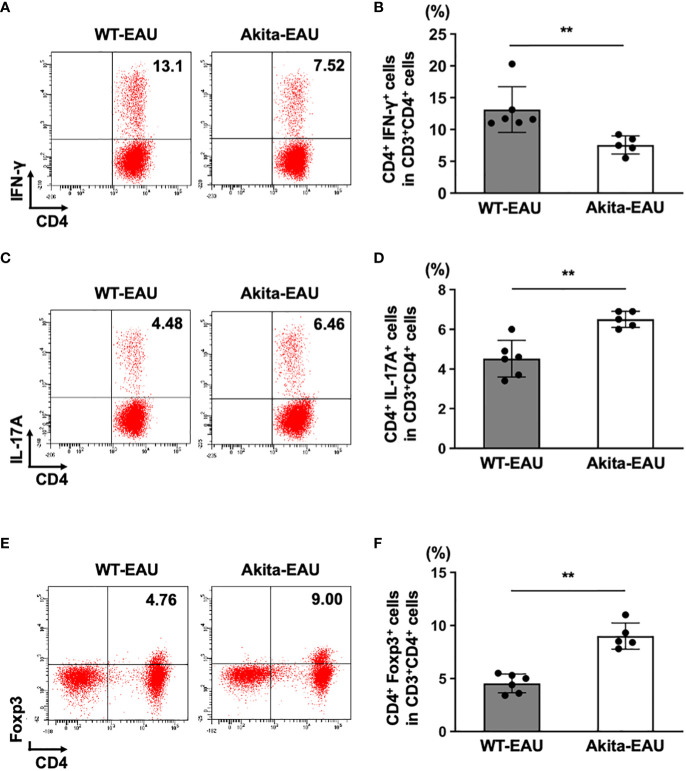
Frequencies of Th1, Th17, and Treg cells in the spleen of Akita mice immunized with hIRBP-p. Spleen cells from immunized WT and Akita mice on day 21 post-immunization were harvested. **(A)** Representative dot plot data for IFN-γ^+^ CD4^+^ T (Th1) cells in spleen cells from one mouse in each group. **(B)** Mean proportion of Th1 cells in the spleen. **(C)** Representative dot plot data for IL-17A^+^ CD4^+^ T cells (Th17) in spleen cells from one mouse in each group. **(D)** Mean proportion of Th17 cells in the spleen. **(E)** Representative dot plot data for Treg cell population in spleen cells from one mouse in each group. **(F)** Mean proportions of Treg cells in the spleen. Data are expressed as mean ± SD for n = 5-6 mice in each group. **P < 0.01 determined using Wilcoxon test. Data are representative of three independent experiments with similar results.

Th1 and Th17 cells play an important role in the pathogenesis of EAU (17, 21), while Treg cells are involved in the remission (30). Since EAU was suppressed in Akita-EAU mice compared with WT-EAU mice despite increased T cell activation in Akita-EAU mice, we examined the proportions of Th1, Th17, and Treg cells in the spleen of WT-EAU and Akita-EAU mice. The spleen cells collected from WT-EAU and Akita-EAU mice on day 21 after immunization with hIRBP-p, and the proportions of CD4^+^CD3^+^ cells producing IFN-γ or IL-17 and CD4^+^Foxp3^+^ (Treg) cells were assayed by flow cytometry (**Figure 4**). The mean proportion of CD4^+^IFN-γ^+^ cells was significantly higher in WT-EAU mice (13.1 ± 3.6%) than in Akita-EAU mice (7.56 ± 1.42%) (**Figures 4A, B**), while the proportion of CD4^+^IL-17^+^ cells and CD4^+^Foxp3^+^ cells were significantly higher in Akita-EAU mice (6.50 ± 0.41% and 9.00 ± 1.24%) than in WT-EAU mice (4.52 ± 0.92% and 4.53 ± 0.88%) (**Figures 4C–F**).

### Antigen-specific cytokine production by T cells in hIRBP-p-immunized Akita mice

**Figure 5:**
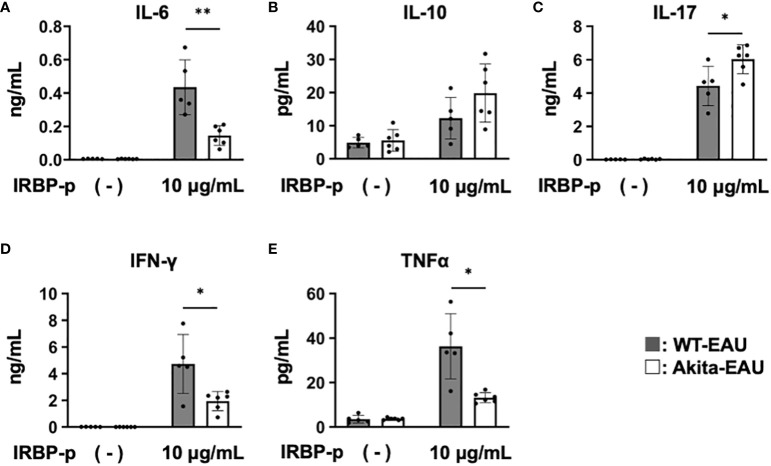
Comparison of cytokine profiles of T cells between WT-EAU and Akita-EAU mice. Splenic T cells were collected from WT-EAU (n = 5) and Akita-EAU mice (n = 5) on day 21-post immunization and cultured with 0 or 10 µg/mL IRBP-p for 72 h. Cytokines in the culture supernatants were measured by Bio-Plex Multiplex Immunoassay System. Mean concentrations of cytokines in culture supernatants of each group for **(A)** IL-6, **(B)** IL-10, **(C)** IL-17, **(D)** IFN-γ, and **(E)** TNFα are shown. Data are expressed as mean ± SD. *P < 0.05, **P < 0.01 determined using Wilcoxon test. Data are representative of three independent experiments with similar results.

Subsequently, to investigate antigen-specific cytokine production by T cells in Akita-EAU mice, splenic T cells from WT-EAU and Akita-EAU mice were cultured with hIRBP-p for 72 hrs, and the cytokines secreted in the supernatants were measured. As shown in **Figure 5**, IL-6, IFN-γ, and TNFα production was significantly higher in WT-EAU mice than in Akita-EAU mice (0.43 ± 0.16 ng vs. 0.14 ± 0.06 ng/ml, 4.73 ± 2.21 ng/ml vs. 1.94 ± 0.71 ng/ml, and 36.3 ± 14.6 pg/ml vs. 13.2 ± 2.2 pg/ml, respectively). However, IL-10 and IL-17 production were conversely higher in Akita-EAU mice (19.8 ± 8.8 pg/ml and 6.02 ± 0.87 ng/ml) compared with WT-EAU mice (12.3 ± 6.3 pg/ml and 4.43 ± 1.18 ng/ml), and there was significant difference in IL-17 production between them.

### Identification of transcriptional changes in Akita mice immunized with hIRBP-p by scRNA-seq

**Figure 6:**
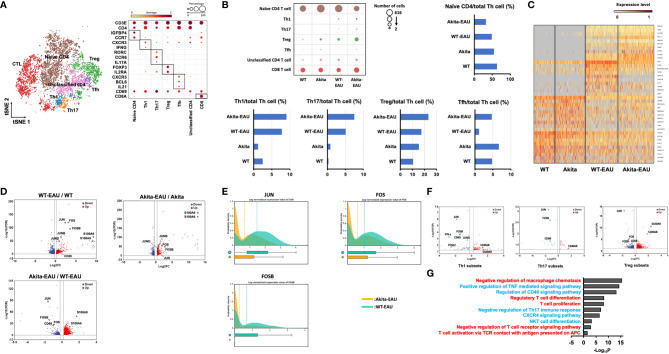
Identification of transcriptional changes associated with Akita-EAU by scRNA-seq. **(A)** t-SNE clustering plot of whole CD3^+^ cells obtained from WT, Akita, WT-EAU, and Akita-EAU mice annotated by immune cell marker gene expression and a bubble heatmap showing percentages of immune cell marker gene expression in individual T cell subsets. **(B)** Bubble heatmap showing number of cells in each T cell subset of four groups and bar graphs showing the proportion of immune cell types. **(C)** A heatmap with hierarchical clusters showing top 39 DEGs for CD3^+^ cells in 4 groups. **(D)** Volcano plot showing up- and downregulated DEGs of Th cells in the comparison of WT-EAU/WT, Akita-EAU/Akita, and Akita-EAU/WT-EAU mice. Red and blue dots indicate up- and downregulated DEGs, respectively. **(E)** Probability density histograms and box plots of JUN, FOS, and FOSB of Th cells in the comparison of Akita-EAU/WT-EAU mice. **(F)** Volcano plot showing up- and downregulated DEGs of Th1 cells, Th17 cells, and Treg cells in the comparison of Akita-EAU/WT-EAU mice. Red and blue dots indicate up- and downregulated DEGs, respectively. **(G)** Major GO analysis of up- and downregulated DEGs of Th subsets in the comparison of Akita-EAU/WT-EAU mice. Red and blue indicate up- and downregulated GO terms, respectively. Each group consisted of 5 mice. *P* value was derived by a hypergeometric test.

In order to elucidate the potential signaling mechanisms underlying the inhibition of EAU in Akita-EAU mice, splenic T cells extracted from WT, Akita, WT-EAU, and Akita-EAU mice were applied for scRNA-seq analysis. As shown in **Figure 6A**, t-SNE clustering plot of whole CD3^+^ cells obtained from 4 groups were annotated to naïve CD4^+^ T cells, Th1 cells, Th17 cells, Treg cells, follicular helper T (Tfh) cells, unknown CD4^+^ T cells, and CD8^+^ cells (CTL) based on marker genes (CD3e, CD4, Insulin-like Growth Factor Binding Protein 4 (IGFBP4), CCR7, CXCR3, IFN-g, RAR-related orphan receptor C (RORC), CCR6, IL-17A, Foxp3, IL-2RA, CXCR5, BCL6, IL-21, CD69, and CD8a). The proportion of naïve T cells was highest in WT mice, followed by Akita mice, WT-EAU mice, and Akita-EAU mice respectively, and differentiated Th cells increased in both WT and Akita mice by immunization with hIRBP-p (**Figure 6B**). The proportion of Th17, Treg, and Tfh cells was higher in Akita mice than in WT-EAU mice, and the difference in the proportion of Th17, Treg, and Tfh cells between them was not affected by immunization with hIRBP-p. In hierarchical cluster analysis performed with the top 39 DEGs for CD3^+^ cells in WT, Akita, WT-EAU, and Akita-EAU, DEGs were broadly classified into four principal clusters (**Figure 6C**). Twenty DEGs from the top row were upregulated in WT-EAU or Akita-EAU mice compared to WT or Akita mice. The first 10 DEGs were primarily more abundant in Akita-EAU mice than WT-EAU mice, while the next 10 DEGs were conversely more abundant in WT-EAU mice. Subsequently, the DEGs from the 21st to the 30th line were downregulated in WT-EAU or Akita-EAU mice compared to WT or Akita mice. The last DEGs from line 31 to 39 were downregulated in other mice compared to WT mice. When differentially expressed genes (DEGs) for Th cells were compared between WT-EAU and WT mice or between Akta-EAU and Akita mice, JUN, FOS, and FOSB, components of the AP-1 signaling pathway, and S100A8 and S100A9, which are involved in the innate immune response, were upregulated in both WT-EAU and Akita-EAU mice (**Figure 6D**). In a comparison of DEGs between WT-EAU and Akita-EAU mice, S100A8 and S100A9 were upregulated, while JUN, FOS, and FOSB were downregulated in Akita-EAU mice compared to WT-EAU mice (**Figures 6D, E**). Significant upregulation of S100A8 and S100A9 and downregulation of JUN, FOS, and FOSB were consistently observed in Th1, Th17, or Treg subsets of Akita-EAU mice compared with WT-EAU mice (**Figure 6F**). **Figure 6G** shows GO analysis performed with up- and downregulated DEGs for Th subsets in the comparison between WT-EAU and Akita-EAU mice. Macrophage chemotaxis, the TNF-mediated signaling pathway, the CD40 signaling pathway, Th17 immune response, the CXCR4 signaling pathway, NKT cell differentiation, and the T cell receptor signaling pathway were downregulated, while regulatory T cell differentiation, T cell proliferation, and T cell activation via TCR contact with antigen presented on APC were upregulated.

### Comparison of JUN and FOS in mRNA expression and secreted protein level by splenic T cells between WT-EAU and Akita-EAU mice

**Figure 7:**
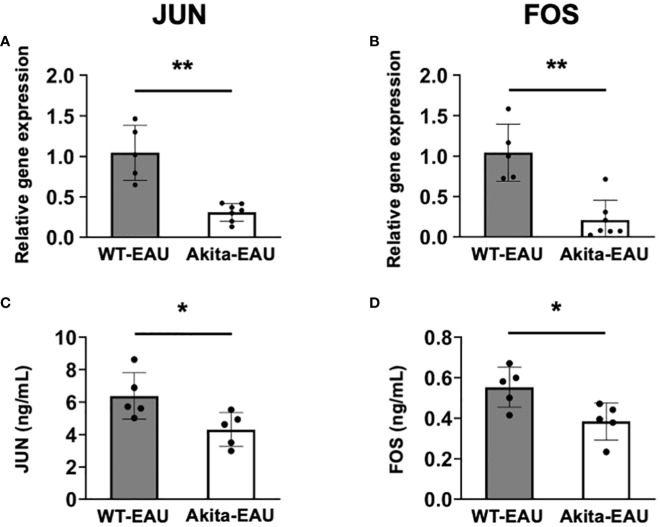
mRNA expression of JUN and FOS in splenic T cells of Akita mice. mRNA expression of JUN **(A)** and FOS **(B)** were analyzed by RT-PCR and the protein levels of JUN **(C)** and FOS **(D)** were measured by ELISA using splenic total T cells collected from WT-EAU (n = 5) and Akita-EAU mice (n = 5) on day 21-post immunization. Data are expressed as mean ± SD. * *P* < 0.05 and **P < 0.01 determined using Wilcoxon test. Data are representative of three independent experiments with similar results.

JUN and FOS are transcription factors involved in T cell differentiation and activation. To further investigate the molecular mechanism underlying in Akita-EAU mice, mRNA expression of JUN and FOS and the protein secretion were compared between WT-EAU and Akita-EAU mice using splenic total T cells. As shown in **Figure 7**, mRNA expression of JUN and FOS in splenic T cells was significantly lower in Akita-EAU mice compared with WT-EAU mice (**Figures 7A, B**). Similarly, the protein concentrations of JUN and FOS secreted from splenic T cells were significantly lower in Akita-EAU mice compared to WT-EAU mice (**Figures 7C, D**).

## Discussion

DM exerts a profound impact on a wide spectrum of tissues, including the eye. This multifaceted disease triggers a cascade of pathophysiological alterations, leading to vascular dysfunction and delayed tissue healing due to compromised local and systemic immune responses. While the detrimental effects of DM on immune cell function have been extensively documented, the underlying mechanisms driving the development of autoimmune diseases, particularly in the ocular compartment, remain incompletely understood. To unravel the intricate interplay between DM and autoimmune pathogenesis, we sought to compare the progression of EAU induced by immunization of hIRBP-p between WT and Akita mice.

EAU is a T-cell mediated antigen-specific autoimmune disease, whereas antigen-presenting cells (APCs) related to innate immunity, such as macrophages and dendritic cells, play a pivotal role to activate and stimulate T cells specific for a retinal antigen in induction phase of EAU. Hyperglycemia is known to affect the number, phenotype, and function of APCs (31–33). If the abilities of APCs are impeded in Akita mice, T cells would not be activated by immunization with hIRBP-p and fail to produce cytokines by stimulation with hIRBP-p. The results that naïve T (CD44^-^CD62L^+^) cells decrease and activated T (CD44^+^CD62L^-^) cells increase in Akita mice by immunization with hIRBP-p, which were significantly more than that of WT mice, indicated that the functions of APCs were preserved and were conversely considered to be enhanced. In contrast, differentiation into Th1 cells and production of IL-6, IFN-γ, and TNFα by T cells in response to hIRBP-p stimulation *in vitro* were significantly lower in Akita-EAU mice compared to WT-EAU mice. This aligns with the finding that EAU incidence and severity were markedly suppressed in Akita-EAU mice compared to WT-EAU mice. These results suggest that diabetic state preserves or promotes the induction phase of EAU, but prevents its onset.

On the other hand, differentiation into Th17 and Treg cells and production of IL-17 by T cells in response to hIRBP-p stimulation *in vitro* were significantly higher in Akita-EAU mice compared to WT-EAU mice. As well in T cells from the DLNs, Th17 and Treg cell differentiation by hIRBP-p immunization was not inhibited in Akita mice compared to WT mice, and Treg cell differentiation was promoted in Akita mice, similar to splenic T cells (**Supplementary Figure S3**). Qiu et al. have reported that mRNA expression in the pancreas and serum level of IL-17A increase in Akita mice compared with WT mice and diabetic signs, such as hyperglycemia, hypoinsulinemia, and inflammatory response, are alleviated in Akita IL-17A deficient mice (34). These results indicated that differentiation into Th17 cells is promoted under diabetic state in Akita mice, which promotes progression of diabetes. The imbalance of Th17 cells and Treg cells is associated with the development of uveitis and other autoimmune and inflammatory diseases (27, 30, 35–43), as evidenced by the expansion of Th17 cells accompanied by a decrease in number or function of Treg cells (44–47). Studies for Treg cells using non-obese diabetic (NOD) mice varied, with some reporting a decrease in Treg frequency and function and others showing no change (48–51). In streptozotocin (STZ)-induced diabetic mice, Muller-Graff et al. reported an increase in Treg cells, and attenuation of autoimmune pancreatitis (52). Zhen et al. also indicated the elevated frequency of Treg cells in STZ-induced diabetic mice, however immune suppressive function of the elevated Treg cells was reduced (53). Unfortunately, there are no reports yet on the frequency and function of Treg cells in Akita mice.

Although both Th1 and Th17 cells are involved in the development of EAU, in Akita-EAU mice, the number of Th17 cells producing IL-17A increased, while the number of Th1 cells producing IFN-γ decreased. In most of previous reports, suppression of EAU is associated with downregulation of both Th1 and Th17 cells. However, Tofacitinib, a JAK inhibitor, suppresses cytokine signaling and thereby inhibits EAU development and IFN-γ production, but has little effect on Th17 or Treg cell production (54). In addition, preimmunization with the B subunit of Escherichia coli heat-labile enterotoxin (EtxB) prevents EAU induction by inhibiting Th1 responses but not Th17 cells (55). On the other hand, Chong et al. reported that Th17 cells are regulated by the production of IL-17A in an autocrine manner, which feeds back to control the Th17 response by inducing the inhibitory cytokine IL-24 (56). Although we have not measured IL-24 production by Th17 cells in Akita-EAU mice, the possibility that the pathogenicity of Th17 cells in Akita-EAU mice is controlled by IL-24 in a suppressive manner cannot be ruled out, since the enhancement of Th17 cell differentiation in Akita-EAU mice was assessed by IL-17A expression.

By utilizing scRNA-seq, we could identify alterations in the composition of immune cell types and gene expression profiles of relevant immune functions of T cells during progression of EAU induced in Akita mice. The proportion of Th17, Treg, Tfh cells was higher in Akita-EAU mice than in WT-EAU mice, which were consistent with the results of increase of CD44^+^CD62L^-^ T cells, Th17 cells, and Treg cells in the other experiments. In comparison of DEGs for Th cells between WT-EAU and Akita-EAU mice, downregulation of JUN, FOS, and FOSB associated with AP-1 signaling pathway in Akita-EAU mice were observed not only in Th1 subset but also in Th17 and Treg subsets. This result suggests that attenuated development of EAU in Akita mice would not result from immunosuppressive roles of Treg cells.

AP-1 is a transcription factor that is consisted of JUN and FOS subunits and regulates gene expression in response to various stimuli, such as growth factors, cytokines, and stress signals, including nuclear gene expression during T-cell differentiation and activation (57–59). Binding of the NF-AT-AP-1 complexes to the target region induces T cell activation. The involvement of AP-1 in the pathogenesis of rheumatoid arthritis and multiple sclerosis has been reported (60–62). Research focusing on endotoxin-induced uveitis (EIU) has revealed that suppression of the AP-1 signaling pathway leads to the inhibition of EIU (63–65). In EAU, downregulation of the AP-1 signaling pathway in Th17 cells and Treg cells has been observed in a study of EAU ameliorated by progesterone (66). However, AP-1 is an upstream transcription factor activated by diverse growth factors and inflammatory cytokines, and has been implicated in T cell responses. In fact, GO analysis indicated that downregulation of the related macrophage chemotaxis, the TNF- and the CD40-mediated signaling pathway, Th17 immune response, the CXCR4 signaling pathway, NKT cell differentiation, and the T cell receptor signaling pathway. Therefore, we have not yet clarified at which stage of the downstream AP-1 signaling pathway in T cells is inhibited under the diabetic environment. We are undertaking adoptive transfer experiments with T cells from WT-EAU to Akita mice and from Akita-EAU mice to WT mice, which allow us to assess whether effector T cells (Th1/Th17) and Tregs cells are functionally impaired or exhibit dysfunctional differentiation in the diabetic environment.

S100A8 and S100A9 are calcium-binding proteins that are expressed by a variety of immune cells, including neutrophils, monocytes, and dendritic cells (67). They are involved in a number of inflammatory processes, including the recruitment of immune cells, the production of inflammatory mediators, and the destruction of tissue. It is reported that S100A8 and S100A9 levels in the blood of T2DM patients are associated with the severity of DR (67). Therefore, upregulation of S100A8 and S100A9 in Th cells of Akita-EAU mice compared to WT-EAU mice in DEGs suggests that EAU and DR may have a synergistic effect.

Tfh cells are a subset of Th cells that are specialized for B cell help. They are characterized by the expression of BCL2 and CXCR5 and are located primarily in the germinal centers of secondary lymphoid organs, such as lymph nodes and the spleen (68). These cells play a crucial role in assisting B lymphocytes in the production of antibodies, thereby contributing to humoral immunity (68). Unlike Th cells or CTLs, Tfh cells lack the ability to function as effector T cells. In transcriptional study using scRNA-seq, frequency of Tfh cells was higher in Akita mice than in WT mice. In addition, Tfh cells increased in response to immunization with hIRBP-p, which was more dominant in Akita mice than in WT mice. IL-21, an autocrine cytokine primarily produced by Tfh and Th17 cells, plays a key role in the immune system (69). It promotes the proliferation and development of Tfh and Th17 cells, maintains the balance of helper T cell subsets, induces B cell generation and differentiation into plasma cells, and enhances immunoglobulin production. In mouse models of diabetes, blocking IL-21 signaling protected against the development of the disease (70). Conversely, transgenic IL-21 expression in pancreatic islets induced diabetes in non-autoimmune C57BL/6 mice (71). Th cells producing IL-21 and the serum level also augment in Type 1 DM patients (72, 73).

In conclusion, EAU was induced in Akita mice by immunization with hIRBP-p, however the severity was significantly lower than that of WT mice. T cells were fully activated in Akita-EAU mice, and the differentiation into Th17 cells was promoted, whereas Th1 cell differentiation was inhibited in Akita-EAU mice. In addition, gene expression associated AP-1 signaling pathway was downregulated not only in Th1 cells but also in Th17 and Treg cells in Akita-EAU mice at the onset of EAU. These findings offer a novel perspective on how DM-induced immune system modulation influences autoimmune conditions.

## Data availability statement

The scRNA-seq datasets in this paper have been deposited in the DNA Data Bank of Japan (DDBJ) Sequence Read Archive (DRA) database under the accession number PRJDB17203. The individual sample accession numbers are as follows: WT: DRR536237, Akita: DRR536238, WT-EAU: DRR536239, Akita-EAU: DRR536240.

## Ethics statement

The animal study was approved by The Animal Ethics Committee of the National Defense Medical College. The study was conducted in accordance with the local legislation and institutional requirements.

## Author contributions

MT: Funding acquisition, Investigation, Methodology, Writing – original draft, Writing – review & editing. YN: Data curation, Investigation, Writing – original draft. HS: Data curation, Investigation, Methodology, Writing – review & editing. TS: Data curation, Formal analysis, Writing – review & editing. AY: Formal analysis, Funding acquisition, Project administration, Validation, Writing – review & editing. MI: Conceptualization, Validation, Writing – review & editing. KH: Data curation, Investigation, Writing – review & editing.
