# Supplementary figures and images for: Autoimmune uveitis attenuated in diabetic mice through imbalance of Th1/Th17 differentiation via suppression of AP-1 signaling pathway in Th cells
Source: Front Immunol. 2024 Jun 3;15:1347018. doi: 10.3389/fimmu.2024.1347018 (PMC11180723; doi:10.3389/fimmu.2024.1347018)

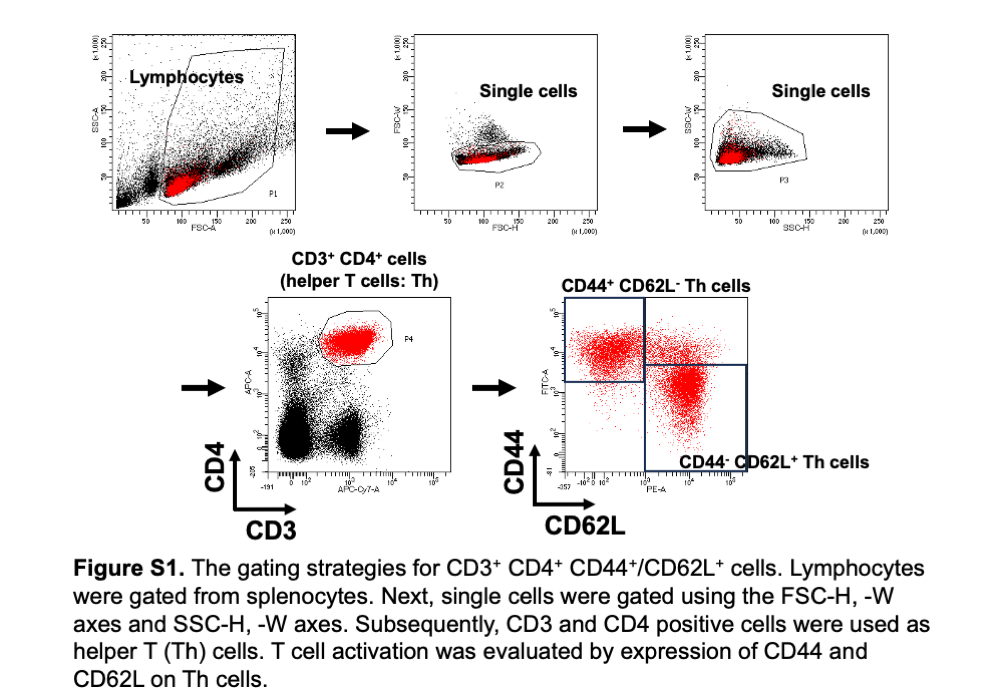

Supplement: Supplementary file 1 [file Image_1.tiff]

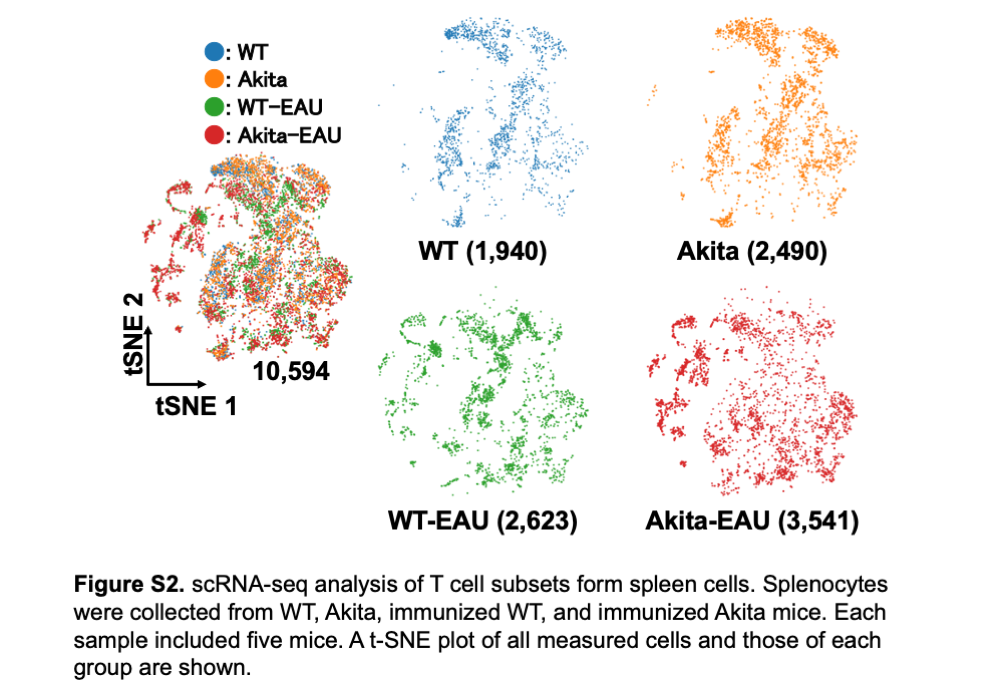

Supplement: Supplementary file 2 [file Image_2.tiff]

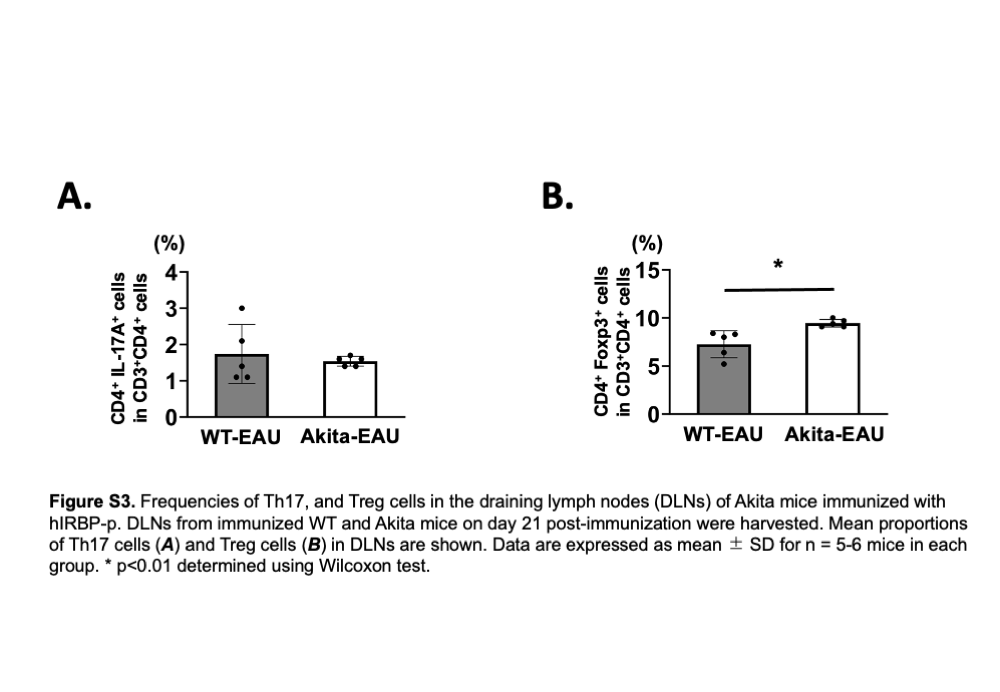

Supplement: Supplementary file 3 [file Image_3.tiff]
